# Effect of yerba mate and green tea on paraoxonase and leptin levels in patients affected by overweight or obesity and dyslipidemia: a randomized clinical trial

**DOI:** 10.1186/s12937-018-0426-y

**Published:** 2019-01-19

**Authors:** Guilherme Balsan, Lúcia Campos Pellanda, Grasiele Sausen, Thaís Galarraga, Denise Zaffari, Bruna Pontin, Vera Lúcia Portal

**Affiliations:** Institute of Cardiology, University Cardiology Foundation – IC/FUC, Avenida Princesa Isabel, 370/ 3 andar., Porto Alegre, Rio Grande do Sul 90620-000 Brazil

**Keywords:** Yerba mate, Green tea, Dyslipidemia, Obesity, Paraoxonase-1 (PON-1), Leptin

## Abstract

**Background:**

This study aimed to evaluate the effect of the intake of yerba mate (YM) and green tea (GT) on serum levels of leptin and paraoxonase-1 (PON-1), compared to control.

**Methods:**

Controlled, randomized clinical trial (RCT) that evaluated 142 men and women affected by overweight or obesity aged 35–60 years, untreated dyslipidemia and no history of coronary artery disease. Participants were randomized to ingest 1000 mL GT, YM or apple tea (AT, control group) daily, during eight weeks. Serum PON-1 and leptin levels were analyzed by ELISA immunoassay at the beginning (baseline) and after eight weeks of intervention.

**Results:**

The intake of 1 l of YM/day resulted in significant increase in serum levels of PON-1 (9.7%; *p* = 0.005). The consumption of GT induced no significant difference in the levels of PON-1 (*p* = 0.154) and leptin (*p* = 0.783). Intergroup analysis showed a significant difference (*p* = 0.036) in the variation of PON-1 levels in the YM group when compared to GT and AT groups. In addition, the increase in PON-1 levels in the YM group was significantly associated with increased HDL-c (*p* = 0.004).

**Conclusions:**

The intake of yerba mate increased the antioxidant capacity by increasing serum levels of PON-1 and was positively associated with increased HDL-c, stressing the protective role of this beverage against atherosclerotic diseases. GT intake had no significant effect on serum levels of PON-1 and leptin.

**Trial registration:**

This study is registered with *ClinicalTrials.gov* under protocol number *NCT00933647*.

## Introduction

The inflammatory process is implicated in the etiology of cardiovascular diseases, responsible for approximately 17 million deaths annually worldwide, representing an increase of 40.8% since 1990 [1, 2]. Oxidative stress is a causal factor, since oxidation of low density lipoprotein (LDL) is linked to the pathogenic processes of dyslipidemia and atherosclerosis [3]. In this context, it has been suggested that the inverse relationship between the intake of phenolic compounds and atherosclerosis observed in epidemiological studies can be partly explained by the antioxidant capacity of phenolic compounds [4, 5].

Beverages prepared with yerba mate (YM) are a potential source of phenolic compounds, particularly phenolic acids, caffeoyl derivatives such as chlorogenic acid (GCA) and some flavonoids such as rutin, quercetin, kaempferol and luteolin, which contribute to a hypocholesterolemic effect and weight loss [6]. It is estimated that YM is consumed by approximately one million people around the world [7, 8].

Green tea (GT) (*Camellia sinensis*) is one of the world’s oldest beverages and also one of the most studied, especially with regard to its role in the prevention of coronary heart disease. Clinical and epidemiological evidence suggests that the intake of GT is associated with improvement of the lipid profile and a decrease in body weight, and could be a strategy to reduce the inflammatory process related to the disease [9–11]. The content of polyphenols present in the drink, especially epigallocatechin-3-gallate, appear to be the main factor responsible for this effect. However, most of the studies have been conducted on eastern populations, where the intake of GT is a ritual of great social and cultural importance [12, 13].

Several studies have shown the antioxidant potential of aqueous extracts of yerba mate [7, 14, 15] and green tea [16–18]. However, few studies have evaluated markers of inflammation such as paraoxonase-1 (PON-1), which is an antioxidant enzyme closely related to high density lipoprotein (HDL), or leptin which is involved in the mechanism of satiety and has increased levels in individuals affected by overweight or obesity [14, 19, 20]. A pilot study with four participants showed increased activity of plasma PON-1 in healthy individuals after acute ingestion of yerba mate extract [21]. There were no studies in the literature that evaluated the effect of yerba mate on leptin in humans, only in animals by assessing gene expression [22–24]. The few studies that have investigated the effect of GT on PON-1 showed a beneficial effect on enzyme levels [25, 26]. On the other hand, several animal studies indicate a beneficial effect of GT on leptin, but studies in humans did not show significant differences [27, 28].

The present study aimed to assess, through a controlled randomized clinical trial (RCT), if the intake of yerba mate can contribute to the improvement of the levels of leptin and PON-1 in individuals affected by overweight or obesity and dyslipidemia, comparing its effects with GT which is already recognized for its cardioprotective benefits.

## Methods

### Outline of the study

Controlled randomized clinical trial with administration of green tea, yerba mate and, apple tea (control group) in subjects affected by overweight or obesity and dyslipidemia.

### Calculation of sample size

The first sample calculation was based on PON-1 activity levels reported by Menini et al. (2007), since there were no previous studies on the effect of green tea and yerba mate on serum levels of PON-1 and leptin in humans [21]. The sample size was estimated at 75 individuals, who were divided into three groups, with an expected difference in increase of serum levels of 10%, considering an alpha of 0.05 and power of 80%. In the initial statistical analysis, the average level of PON-1 presented an important increase of 4% (92 U/mL) among the groups, with *p* = 0.09. The sample size was recalculated, based on these results, showing the need for 45 individuals per group, with an expected intergroup difference of 8% (200 U/mL), considering an alpha of 0.05 and a power of 70%. For the analysis of PON-1 and leptin levels, 142 frozen serum samples were used, randomly distributed in groups YM (*n* = 47), GT (*n* = 49) and AT (*n* = 46) by researchers who were not part of the study.

### Selection of participants

Participants were recruited through public calls between November 2007 and September 2009. The inclusion criteria used were: men and women 35 to 60 years of age, affected by overweight or obesity, dyslipidemic, and without previous diagnosis of cardiovascular disease.

Exclusion criteria were: presence of neoplasms, infections and hepatic, renal or gastrointestinal conditions; levels of triglycerides (TG) > 400 mg/dL; pregnancy and lactation; alcohol consumption > 4 doses a day; use of estrogen, nonsteroidal anti-inflammatory drugs, antiobesity drugs and vitamin supplementation; use or indication for the use of statins, fibrates and other lipid-lowering drugs; unexplained weight loss (> 2 kg) in the last 30 days; and reported intolerance to any of the beverages evaluated in the study.

The presence of hypertension or diabetes was determined by use of antihypertensive or antidiabetic drugs, respectively, or previous diagnosis of the disease. Dyslipidemia was considered when at least one of the following biochemical parameters was altered: TC > 200 mg/dl and/or TG > 150 mg/dL and/or cholesterol in high density lipoprotein (HDL-c) < 40 mg/dL for men and < 50 mg/dL for women. The LDL-c was calculated by the Friedwald equation and was not the only lipid parameter considered because our interventions could cause changes in both HDL-c and triglycerides. Overweight or obesity was evaluated through the BMI, and included individuals with a BMI between 25 and 35 kg/m^2^ of body surface area. Weight and height were measured using the Welmy LCD W110H*®* anthropometric electronic scale. In order to measure the weight, the participants were instructed to be barefoot, to empty their pockets, to remove adornments and, with the minimum of clothes, being positioned in the center of the digital platform with their arms along the body and hands facing the thighs. Body mass index (BMI) was calculated from weight /height ^2^.

The participants were also asked about alcohol consumption (≥ 30 g de ethanol/day for men and ≥ 15 g of ethanol/day for women) and, smoking (categorized as a smoker, defined as those that smoked one or more cigarettes a day, ex-smoker or non-smoker).

Subjects who performed physical activity more than 30 min per session at least three days a week were considered physically active. We considered sedentary, those without structured and routine physical activity, performed less than three days a week, < 30 min per session.

The project was approved by the Research Ethics Committee of Institute of Cardiology of Rio Grande do Sul - University Foundation of Cardiology (IC/FUC), and all participants signed an informed consent form.

### Study protocol

During the first consultation (week - 4), a standardized questionnaire was applied to the patients and weight and height were measured. After a 12-h fasting, the participants were forwarded to the laboratory of Institute of Cardiology of Rio Grande do Sul for biochemical tests. Participants meeting the inclusion criteria and no exclusion criteria were then instructed to not ingest green tea, yerba mate, mate tea, apple tea or any other kind of tea for 4 weeks (run-in period), and to maintain their usual lifestyle.

After the run-in period, participants were randomly allocated to the green tea, yerba mate or apple tea groups. Apple tea was chosen as control based on the study of Lima et al. (2004) that showed that this was the Brazilian tea with the lowest content of polyphenols [29].

Block randomization was generated by an outside researcher and opaque, sealed and numbered envelopes were used to ensure blind allocation. At this point (week 0), participants had their anthropometric measurements taken and received verbal and printed orientation on how to ingest the appropriate tea. In addition, they were instructed to maintain their usual dietary and physical activity habits during the study. Participants assigned to the yerba mate group were asked to prepare the drink in a standardized gourd recipient, using 87.5 g (approximately 15 tablespoons) of yerba mate and 500 mL of hot water. Two gourds should be prepared during the day, with a total intake of 1000 mL of mate. The participants were also instructed not to share their drink, so that the total volume was taken only by them. The participants of the green tea and apple tea groups were asked to prepared the infusion using a sachet of 1 g of tea for each 200 mL of hot water, five times a day, totaling a volume of 1000 mL. The recommended infusion time was 3 min, and the temperature of the water should be around 70 °C. Other substances such as sugar, honey, dried fruits, herbs and other teas should not be added to the tea, but artificial sweeteners were allowed. On week 0, the participants received kits of yerba mate (6 kg of yerba mate, one standard gourd and one bombilla), green tea (140 sachets) and apple tea (140 sachets), to be consumed over a period of four weeks. The participants were instructed not to consume the other two types of teas included in the study, or any other types of tea. In week 4, the participants returned to the clinic to receive a new kit. Finally, in week 8, after a 12-h fasting the participants underwent a new biochemical and anthropometric assessment. The physical conditions and any adverse effects were examined and registered during consultations (weeks 4 and 8). Any leftover tea and yerba mate were returned and recorded in medical records, for analysis of adherence to the treatment. Participants with altered lipid parameters at the end of the study were instructed to consult a cardiologist.

### Analysis of PON-1 and leptin

Serum samples were analyzed by ELISA for quantification of PON-1 and leptin levels. Commercial kits were used (Quantikine, R&D Systems and/or Mercodia), and absorbance was analyzed by spectrophotometry (Spectramax M2, Molecular Devices) with quantification through the computer program SoftMaxPro (Molecular Devices). Interpolations were made from a regression curve of the standard protein with the program Excel. The researchers were blinded to the groups (mate, green tea or placebo).

### Registration in ClinicalTrials.gov

This study is registered with *ClinicalTrials.gov* under protocol number *NCT00933647*.

### Statistical analysis

Data were analyzed with the SPSS statistical program, version 18.0. Variables are presented as absolute and relative frequencies, mean ± standard deviation or median and interquartile range (25 and 75 percentile). The statistical tests of analysis of variance (*ANOVA*), Kruskal-Wallis and Pearson’s Chi-square were used to assess the difference of the groups at baseline characteristics. Intra-group differences were assessed with the method of generalized estimating equations with multiple comparisons adjusted by Bonferroni method. Between group variations (deltas; Δ) in the main outcomes (PON and leptin) were estimated by analysis of covariance (*ANCOVA*), to adjust basal values. The relationship of the variables under study with the serum level of the enzymes, according to the group, was investigated with the Pearson correlation coefficient test. Multiple linear regressions were then used to evaluate the relationship of the enzyme levels with lipid and inflammatory markers in the yerba mate group (which showed significant differences in the analyses described above). Values of *p* < 0.05 were considered statistically significant.

## Results

**Fig. 1:**
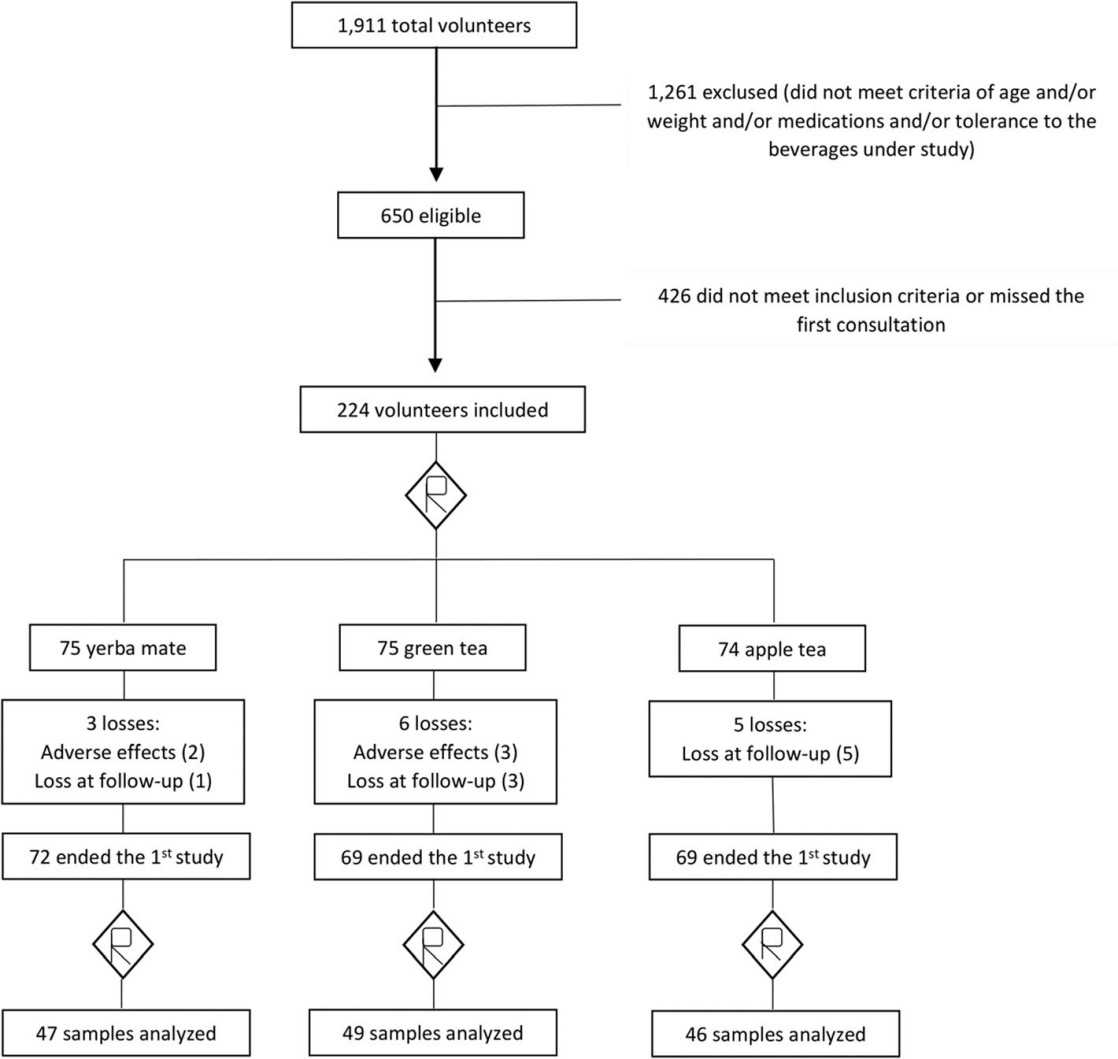
Flow chart of patients

Figure 1 shows the random distribution and the number of participants in the groups. The levels of PON-1 and leptin were determined in 142 serum samples, including 47 samples from the yerba mate group, 49 of the green tea group and 46 from the apple tea group.

The mean age of the participants was 50 years old, and they were mostly females (61%; *n* = 87). Baseline characteristics were similar between the groups, as presented in Tables 1 and 2 that show respectively the basal and anthropometric parameters, and lipid and inflammatory parameters in the three groups.

**Table 1:** Baseline characteristics of participants in the study

Variables	Yerba mate group	Green tea group	Control group	*P*
	Mean ± SD	Mean ± SD	Mean ± SD	
Age (years)	50.3 ± 6.3	49.8 ± 6.7	50.4 ± 6.2	0.879*
Gender – *n* (%)				0.744**
Male	20 (42.6)	19 (38.8)	16 (34.8)	
Female	27 (57.4)	30 (61.2)	30 (65.2)	
Weight	78 ± 11.7	80 ± 11.4	78 ± 10.1	0.250*
BMI	28 ± 2.3	29 ± 2.9	29 ± 3.2	0.374*
WC	92 ± 7.7	93 ± 8.3	91 ± 8.3	0.370*
AC	99.1 ± 6.3	101.3 ± 7.8	78 ± 7.5	0.246*
Risk factors – *n* (%)				
DM	1 (2.1)	2 (4.1)	1 (2.2)	0.804**
HAS	14 (29.8)	12 (24.5)	14 (30.4)	0.777**
Smoking	7 (14.9)	5 (10.2)	6 (13)	0.785**
Medications – *n* (%)				
Diuretics	4 (8.5)	6 (12.2)	6 (13)	0.760**
Beta-blocker	7 (14.9)	6 (12.2)	6 (13)	0.927**
ACEI	5 (10.6)	6 (12.2)	9 (19.6)	0.419**
Wine intake – *n* (%)	13 (27.7)	9 (18.4)	6 (13)	0.200**

* Data presented as mean ± SD, compared by ANOVA

** Data presented as absolute and relative frequencies, compared by the Chi-square test

Values of *p* < 0.05 were considered statistically significant

Abbreviations: *DM* Diabetes mellitus, *HAS* hypertension, *BMI* body mass index, *WC* waist circumference, *AC* abdominal circumference, *ACEI* angiotensin-converting enzyme inhibitor

**Table 2:** Lipid and inflammatory parameters before the intervention

Variables	Yerba mate group	Green tea group	Control group	*P*
	Mean ± SD	Mean ± SD	Mean ± SD	
Blood glucose (mg/dL)	96 ± 8.8	96.1 ± 14.9	96.5 ± 17.8	0.980*
Triglycerides (mg/dL)	132.6 ± 71.5	154.5 ± 75.5	143.7 ± 77.5	0.341*
Total Cholesterol (mg/dL)	221.4 ± 31.9	223.3 ± 42.6	221.3 ± 31.5	0.952*
HDL-c (mg/dL)	45.1 ± 12.4	44 ± 11.8	44.9 ± 14	0.907*
Non-HDL Cholesterol (mg/dL)	176.2 ± 29.3	179.3 ± 39.3	183.8 ± 25.2	0.878*
LDL-c (mg/dL)	151 ± 26.1	149.4 ± 34	148.3 ± 28.8	0.914*
TC/HDL-c ratio	5.2 ± 1.44	5.3 ± 1.36	5.2 ± 1.3	0.969*
Insulin (U/mL)	8.6 (6.1 a 11.7)	9.1 (5.4 a 13.8)	8.1 (6.4 a 10.7)	0.837**
SGOT (U/L)	9 (8 a 11)	9 (8 a 11)	9 (7.8 a 11)	0.938**
Creatinine (mg/dL)	0.67 (0.57 a 0.81)	0.68 (0.61 a 0.79)	0.67 (0.62 a 0.80)	0.989**
CRP (mg/dL)	0.16 (0.1 a 0.34)	0.21 (0.1 a 0.37)	0.21 (0.11 a 0.42)	0.695**

* Data presented as mean ± standard deviation, compared by ANOVA

** Data presented as median (interquartile range), compared by Kruskall-Wallis test

Values of *p* < 0.05 was considered statistically significant

Abbreviations: *HDL-c* high density lipoprotein cholesterol, *Calculated LDC-c* low-density lipoprotein cholesterol, *SGOT* serum glutamic oxaloacetic transaminase, *CRP* high sensitive C-reactive protein

Other parameters evaluated included thyroid disease, psychological diseases, and use of medications such as calcium channel blockers, centrally acting blockers, angiotensin blockers, antidepressants, benzodiazepines, thyroid hormones, antiplatelet drugs and anticonvulsants. These variables were infrequent and/or statistically similar among groups.

Serum levels of PON-1 presented significant intra-group and intergroup differences, as shown in Table 3. Intergroup analysis showed a significant difference (*p* = 0.036) of the yerba mate group in relation to the other two groups, with a positive variation of 250 U/mL with post-pre-adjusted Δ (95% IC, 57 to 444). The green tea and apple tea groups presented negative variations of − 55 U/mL (95% IC, − 248 to 137) and − 66 (95% IC, − 264 to 132), respectively.

**Table 3:** Mean values and standard deviation of paraoxonase enzymes (PON-1) and Leptin pre- and post-intervention with yerba mate, green tea and apple tea and its gross and adjusted variations between groups for 8 weeks

Variables		Intra-group		P*	Intergroup		*P*
		Pre	Post		Crude post-pre Δ	Adjusted post-pre Δ**	
	*N*	Mean ± SD	Mean ± SD		(95% CI)	(95% CI)	
Paraoxonase							
Yerba mate group	47	2625 ± 1017	2880 ± 912	**0.005**	255 (42 a 468)	250 (57 a 444)	**0.036**
Green tea group	49	2899 ± 786	2745 ± 923	0.154	− 153 (− 361 a 54)	−55 (− 248 a 137)	
Control group	46	2369 ± 675	2403 ± 684	0.629	33 (− 181 a 249)	−66 (−264 a 132)	
Leptin							
Yerba mate group	47	25,010 ± 16,344	24,849 ± 16,226	0.908	− 160 (− 2551 a 2230)	− 0.440 (− 2275 a 2274)	0.450
Green tea group	49	25,049 ± 15,775	25,288 ± 14,551	0.783	238 (− 2104 a 2580)	405 (1823 a 2633)	
Control group	46	22,028 ± 15,296	20,813 ± 15,160	0.347	− 1214 (− 3632 a 1202)	−1556 (− 3860 a 748)	

* Method of generalized estimating equations with multiple comparisons adjusted by Bonferroni method (intra group)

** ANCOVA with adjustment for baseline levels among the 3 groups

Intra-group analysis also showed increase in serum levels of PON-1 in the yerba mate group (Table 3). A comparison of results before and after the intervention showed an increase from 2625 pg/mL to 2880 pg/mL in serum levels (255 pg/mL or 9.7%; *p* = 0.005). On the other hand, a decrease in serum levels of PON-1 was observed in the green tea group, from 2899 pg/mL to 2745 pg/mL (− 154 pg/mL or − 5.3%; *p* = 0.154), although this difference was not significant. Surprisingly, an increase of PON-1 levels was observed in the apple tea group, from 2369 pg/mL to 2403 pg/mL (34 pg/mL or 1.4%; *p* = 0.629), but the difference was not significant.

No significant changes were observed for leptin levels in intra-group (pre and post intervention) (Table 3) and intergroup analyses. However, it is important to note that leptin levels were improved in the yerba mate group (decrease of 161 pg/mL or 0.64%; *p* = 0.980) and apple tea group, with emphasis on the reduction of 1556 pg/mL in the apple tea group (− 5.5%; *p* = 0.347). However, leptin levels were increased from 25,049 pg/mL to 25,288 pg/mL in the green tea group (239 pg/mL or 0,9%; *p* = 0.783), which represented again an effect contrary to the expected.

Table 4 presents the multiple linear regressions of the enzymes PON-1 and leptin with different specific markers considering final levels and the variation for each enzyme before and after the intervention in the yerba mate group, to evaluate the interaction between them. PON-1 levels were evaluated in relation to cholesterol in high density lipoprotein (HDL-c), insulin, cholesterol in the low-density lipoprotein (LDL-c), TG and C-reactive protein (CRP). The results show a significant relationship of the variation of PON-1 levels with HDL-c (*β* = 0.443; *p* = 0.004). PON-1 levels were not associated with final levels of the other markers studied. The enzyme leptin was assessed in relation to HDL-c, TG, insulin, abdominal circumference (AC) and BMI. The results show that post-intervention levels of leptin have no significant relationship with any of the markers. The enzyme leptin was evaluated in relation to HDL-c, TG, insulin, AC and BMI. The only significant differences observed in the analysis of variance were with insulin (*β* = 0.353; *p* = 0.005) and BMI (*β* = 0.554; *p* = 0.001).

**Table 4:** Multiple linear regressions of the PON and leptin enzymes with different specific markers considering the final levels and pre and post intervention variation in the yerba mate group, for each enzyme, in order to evaluate the interaction between them

Enzymes	B	*β*	*P*	R squared
Paraoxonase Post				0.106
HDL-c	16.795	0.242	0.145	
Insulin	−43.694	−0.203	0.193	
LDL-c	−1.784	−0.053	0.741	
Triglycerides	1.992	0.159	0.361	
CRP	29,334	0.007	0.965	
Paraoxonase Delta				**0.243**
HDL-c	62.294	0.443	0.004*	
Insulin	70.899	0.242	0.083	
LDL-c	3.150	0.069	0.620	
Triglycerides	1.318	0.082	0.586	
CRP	− 106.351	−0.052	0.714	
Leptin Post				0.163
HDL-c	218.552	0.177	0.249	
Insulin	700.426	0.183	0.221	
LDL-c	−57.335	−0.096	0.515	
AC	− 642.768	−0.263	0.151	
BMI	2866.538	0.418	0.023	
Leptin Delta				**0.441**
HDL-c	−64.652	−0.043	0.716	
Insulin	1096.008	0.353	0.005*	
LDL-c	−30.036	−0.062	0.610	
AC	34.941	0.010	0.949	
BMI	10,033.978	0.554	0.001**	

N = 47 participants

B: non-standard coefficient of multiple linear regression

Beta: standardized coefficient of multiple linear regression

*P*: value *P*

R squared: coefficient of determination of multiple linear regression

* *p* < 0.05; ** *p* < 0.01

Abbreviations: *HDL-c* high density lipoprotein cholesterol, *Calculated LDC-c* low-density lipoprotein cholesterol, *TG* Triglycerides, *CRP* high sensitive C-reactive protein, *CA* abdominal circumference, *BMI* body mass index

## Discussion

This was the first randomized clinical trial to compare the effects of drinking yerba mate and green tea on serum levels of PON-1 and leptin in individuals affected by overweight or obesity and dyslipidemia. The study showed that the daily intake of 1 l of yerba mate for eight weeks increased the serum levels of PON-1, an enzyme with important anti-inflammatory and antioxidant roles.

The analysis of intergroup variation demonstrated that the yerba mate group presented significant differences of serum levels of the enzyme PON-1 compared to the groups that ingested green tea and apple tea (Table 3).

Although it was not possible to characterize the polyphenol content in the studied yerba mate, the difference found between the studied groups (mate, green tea and apple tea) can be explained by the fact that yerba mate has a high concentration of polyphenols, around 2 to 2.5 times greater than green tea for instance [30, 31]. In addition, the phenolic compounds found in yerba mate are structurally different from those of green tea, with a greater concentration of CGA but not of catechins (main component of green tea) in yerba mate. These differences can interfere directly in the effect of the beverages [30]. Bixby et al. (2005) compared three commonly consumed beverages rich in polyphenols with well-known antioxidant activity: yerba mate, green tea and red and white wines of different varietals. The results observed with the extract of yerba mate, which was prepared in the form usually ingested by the population, were superior to those obtained with all other beverages. In addition, yerba mate presented the greatest concentration of polyphenols, followed by red wine and green tea, as well as a higher activity of chelation of free radicals [32]. Bastos et al. (2007) have also observed higher in vitro antioxidant activity in extracts of yerba mate than green tea (*Camellia sinensis*) [33]. Other studies have indicated that yerba mate has antioxidant activity equivalent to or greater than vitamin C and vitamin E, which are considered as reference for this characteristic. These evidences stress the strong positive influence of yerba mate observed in the present study [34, 35].

In the current study, the daily ingestion of one liter of yerba mate during 4 weeks also induced significant increase (9.7%) of the serum levels of the antioxidant enzyme PON-1 observed by comparing PON-1 levels before and after the intervention (Table 3). This result is in accordance with the preliminary study of Menini et al. (2007), which reported that acute ingestion of yerba mate infusion increased by 10% on average, the PON-1 activity in the plasma of four healthy individuals [21]. Other studies have also indicated a positive effect of the intake of yerba mate on the activity and serum levels of PON-1 [7, 36]. Matsumoto et al. (2009) investigated the effects of mate tea supplementation on plasma susceptibility to oxidation and expression of genes coding for antioxidant enzymes in healthy non-smoking women, after acute or prolonged intake. Lipid peroxidation was greatly reduced after the period of supplementation with yerba mate, an effect that remained after prolonged administration. The total antioxidant capacity and the level of gene expression of the antioxidant enzyme were also improved after the prolonged consumption [37]. Gugliucci et al. (2009) observed that the CGA (main phenolic compound of yerba mate) protects the activity of human PON-1 by inactivation due to physiological concentrations of hypochlorite [36]. Using in vitro analyses (macrophage activity) and in vivo studies with intake of infusions of green or toasted yerba mate by healthy women, Fernandes et al. (2012) showed that yerba mate increased the expression of the gene coding for paraoxonase-2 (PON-1-2) [37]. On the other hand, no increase of paraoxonase-1 (PON-1) was observed by Bonaventure et al. (2012) after long-term ingestion of yerba mate infusion, although an increase around 23% was seen in 50% of the subjects, who were also dyslipidemic [14]. These studies suggest that habitual consumption of yerba mate can increase the body’s antioxidant defense through several mechanisms, and that the plasma antioxidant capacity can be improved even in healthy subjects [38].

Epidemiological and cohort studies provided convincing evidence on the protective paper of PON-1 against artery disease, through its ability to prevent lipid oxidation and limit the development of atherosclerotic lesion due to its connection with HDL [39–41]. In the present study, the variation in the levels of PON-1 also presented significant association (*β* = 0.443; *p* = 0.004) with HDL. This result reinforces the fact that PON-1 is closely associated with HDL, promotes the inhibition of LDL oxidation and decreases oxidized lipids in atherosclerotic lesions. It is also an evidence of the antioxidant activity of yerba mate in humans, resulting in increased levels of the enzyme and a positive interaction with HDL [39, 42]. These results suggest that yerba mate and other nutritional antioxidants, by a mechanism not yet fully elucidated, improve levels of antioxidant enzyme PON-1 and can protect against cardiovascular events. One of the likely mechanisms is the ability to hydrolyze specific oxidized lipids in lipoproteins, macrophages and atherosclerotic lesions [43]. Thus, strategies to promote the increase of PON-1 activity are important to reduce the risk of development of atherosclerosis [43]. Although leptin levels did not show significant differences in intergroup and intra-group analyses, variation on its level was associated with insulin levels (*β* = 0.353; *p* = 0.005) and BMI (*β* = 0.554; *p* = 0.001).

Leptin exerts its effect on the energy balance primarily by acting in the brain. Insulin and BMI are directly related to leptin, which acts directly or by activating specific centers in the hypothalamus to decrease food intake, increase energy expenditure, regulate glucose and fat metabolism, or to change neuroendocrine function [44, 45].

Leptin levels increase exponentially with the increase in fat and in the presence of disturbances caused by increase in visceral adiposity. Much has been done to investigate the mechanisms responsible for these variations [46, 47]. Some proteins present in the adipose tissue and skeletal muscle appear to be related to the mechanisms by which this increase in adipose tissue and in the concentrations of circulating free fatty acids would result in the development of insulin resistance and type 2 diabetes. Individuals with visceral obesity have greater probability of developing insulin resistance and hyperinsulinemia when compared to individuals with other types of obesity [48]. Insulin acts on the uptake of glucose by cells, allowing its conversion into energy or its storage in the form of glucogen [49]. Insulin resistance is characterized by a lower than normal glycemic response to insulin, which can lead to chronic hyperglycemia with disorders of the metabolism of carbohydrates, lipids and proteins. The compensatory metabolic response to insulin resistance is hyperinsulinemia, aiming at the maintenance of blood glucose levels [50, 51].

There is evidence that some polyphenols, especially CGA which is very abundant in yerba mate, have effects on the hepatic mechanism of glucose and also on the pattern of its intestinal absorption [52] Pang et al. (2008) reported that supplementation of animal feed with yerba mate significantly reduced blood glucose and insulinemia in animals with obesity [53]. Oliveira et al. (2008) observed that yerba mate induced a significant decrease in gene expression of glucose cotransporters in the small intestine of both diabetic and non-diabetics animals, suggesting that the bioactive compounds of yerba mate reduce the absorption of glucose [52]. It is known that regular consumption of yerba mate interferes with the absorption of glucose, which could be a further positive factor in its ingestion. However, new studies are still needed to understand this mechanism of action in humans and whether it can effectively lead to blood sugar reduction and increased tolerance to glucose [23].

Considering the association of leptin levels with BMI in the yerba mate group, some epidemiological studies suggest that intake of yerba mate can have positive effect on weight loss [54]. However, the number of controlled, randomized clinical trials is still small and the results are inconsistent. The main effect of leptin is the regulation of adipose tissue and body weight. Firstly, the decrease in appetite and energy consumption of the body are explained by the decrease in secretion of neuropeptide Y (NPY) and by increased secretion of melanocyte-stimulating hormones (MSH-α), resulting from the linking of leptin to its receptor in the hypothalamus. Secondly, the increase in energy consumption is achieved by heat release, which is affected by massive energy storage. The process is due to increased activity of sympathetic nerves induced by leptin and activation of the adrenaline receptor on the membranes of fat cells. Thirdly, the production of fatty tissue is directly influenced by leptin and resorption can be accelerated. Some evidence suggests that leptin can induce the maturation of adipose cells. Finally, leptin might influence other hormones: insulin can accelerate the secretion of leptin and, conversely, leptin has a negative feedback on the synthesis and secretion of insulin. The higher the BMI or waist circumference, the higher is the serum level of leptin [55].

This study presents some limitations, such as: (1) the use of green tea in sachet can have affected, at least partially, the results obtained from that group. It is possible that the polyphenol content of packed tea is lower than in tea prepared from the whole leaf of *Camellia sinensis*, which could reduce its beneficial effects. However, this is the form usually available in Brazil and possibly the most consumed. In addition, (2) a blind study was not possible, since only the use of encapsulated extracts of yerba mate and green tea would have allowed this approach. The intent was to conduct a study of effectiveness and cardioprotective effect of the teas in the way they are popularly consumed. For this same reason, (3) dietary factors were not controlled, except for the orientation of participants to maintain their usual dietary habits and lifestyle.

Further research is required to identify non-pharmacological therapeutic options that provide additional benefits to cardiovascular health. The therapeutic dietary management is a first step in reducing the risk of coronary heart diseases, and the current dietary recommendations suggest the inclusion of food and not their exclusion [3, 19, 22, 23, 35]. The biggest challenge is to integrate this ever-growing list of foods to the diet without, however, increasing energy consumption beyond what is necessary for the acquisition or maintenance of a healthy weight.

## Conclusion

The daily intake of one liter of yerba mate for eight weeks in subjects affected by overweight or obesity and dyslipidemia increased their antioxidant capacity through the elevation of serum levels of PON-1 and associated positively with the increase of the HLD-c, stressing the protective role of this compound against atherosclerotic disease. In addition, the reduction in leptin levels in the YM group was significantly related to reduction of insulin and BMI. The intake of green tea showed no significant effect on serum levels of leptin and PON-1.

These results demonstrate the antioxidant role of yerba mate and its possible benefits in glycemic metabolism and in the control of body weight. Further studies are needed with a greater number of participants to confirm these results.

## Abbreviations

AC: Abdominal circumference
AT: Apple tea
BMI: Body mass index
CRP: C-reactive protein
GCA: Chlorogenic acid
GT: Green tea
HDL: High density lipoprotein
HDL-c: Cholesterol in high density lipoprotein
LDL: Low density lipoprotein
LDL-c: Cholesterol in the low-density lipoprotein
PON-1: Paroxinase-1
RCT: Randomized clinical trial
TC: Total cholesterol
TG: Triglycerides
YM: Yerba mate
